# Review of outcomes of delayed chest closure following lung transplantation: a meta-analysis

**DOI:** 10.1186/s13019-022-01868-w

**Published:** 2022-05-19

**Authors:** Cheng Chen, Quan Zheng, Dongsheng Wu, Yongxiang Song, Gang Xu

**Affiliations:** 1 Department of Thoracic Surgery, Affiliated Hospital of Zunyi Medical University, Zunyi, 563000 China; 2 Department of Thoracic Surgery, West China Hospital, Sichuan University, Chengdu, 610041 China; 3 West China School of Medicine, Sichuan University, Chengdu, 610041 China

**Keywords:** Delayed chest closure, Lung transplantation, Meta-analysis, Primary graft dysfunction, Surgical site infection

## Abstract

**Purpose:**

The clinical outcomes of delayed chest closure (DCC) compared with primary chest closure (PCC) following lung transplantation, including perioperative outcomes and long-term survival, remained controversial. This was the first systematic review and meta-analysis aimed to identify the short- and long-term outcomes of DCC following lung transplantation.

**Methods:**

We comprehensively searched electronic literature from 4 databases up to April 1st, 2022. Dichotomous data and continuous data were pooled with odds ratio and weighted mean difference, respectively. The quality of included studies was assessed with the Newcastle–Ottawa Scale.

**Results:**

Ten studies were included in the systematic review and 4 studies were included in the meta-analysis. Pooled analysis showed that DCC was associated with an increased risk of surgical site infection, prolonged hospital stays, and higher risk of primary graft dysfunction compared to PCC. The 30 day and 5 year survival were higher in PCC cohort compared with DCC cohort while differences in survival at 6 months was insignificant.

**Conclusion:**

Our findings do not support the aggressive application of DCC. DCC should be cautiously applied since its association with worse perioperative outcomes and higher mortality. But it remains the life-saving steps under dangerous circumstances.

## Introduction

Lung transplantation has been the most promising strategy for various end-stage lung diseases, including chronic obstructive pulmonary diseases, idiopathic pulmonary fibrosis, and cystic fibrosis [1]. In the settings of oversized grafts, significant intraoperative injures, hemodynamic instability, pulmonary edema, and coagulopathy, delayed chest closure (DCC) has emerged as an alternative to primary chest closure (PCC) after lung transplantation [2]. DCC might prevent excessive compression on the injured lungs or dilated right ventricle, therefore having the potential to reduce the incidence of primary graft dysfunction (PGD), which was the main cause of postoperative morbidity and mortality after lung transplantation [3, 4].

The need for DCC after lung transplantation ranged from 6 to 29% as published [2, 4–6]. However, the indications and clinical practice guideline of DCC have not been established, and the clinical outcomes remained controversial in the published studies. There have not been any systematic reviews focusing on the clinical outcomes of DCC compared with PCC. Herein, we conducted a systematic review and meta-analysis to identify the short- and long-term outcomes of DCC following lung transplantation.

## Methods

### Data resources

Two independent reviewers retrieved literature from PubMed, OVID, Web of Science, and Cochrane Library from their inception to April 1st, 2022. The search items mainly comprised “delayed chest closure” or “delayed sternal closure” or “open chest management” AND “lung transplantation” or “lung transplant”. The reference lists of included studies were searched manually for any potentially eligible articles. Two reviewers screened potential eligible papers independently, with any disagreement solved by further discussion or arbitrated by a third reviewer.

### Study selection

Inclusion criteria were as follows: (1) Studies with patients who underwent lung transplantation; (2) Studies evaluating the clinical outcomes of DCC; (3) Studies with accessible and essential data. The studies would be excluded if met any of the following criteria: (1) Review, conference abstract, case report or case series, animal experiment, letter, or comment; (2) The article was not written in English; (3) Basic data could not be extracted.

### Quality assessment

We used the Newcastle–Ottawa Quality Assessment Scale (NOS) to assess the quality of each study with three aspects: the selection of patients, comparability of groups, and assessment of outcome [7]. We graded the quality of included studies as “good” (6–9) or “bad” (0–5). Quality assessment was performed independently by two authors.

### Data extraction

Two independent reviewers collected the baseline characteristics from the included studies as follows: the first author, publication year, study design, sample size, country, enrolled year, the number of bilateral lung transplantation, indications for transplantation, type of incision, and indications for DCC. The primary outcome was the incidence of surgical site infection. The secondary outcomes included the incidence of PGD, acute renal insufficiency, postoperative extracorporeal membrane oxygenation (ECMO) use, length of hospital stay, and survival. The definition of PGD followed the current ISHLT guidelines [8]. The renal insufficiency was defined as severe renal dysfunction requiring dialysis treatment.

### Statistical analysis

We conducted the meta-analysis with Review Manager 5.3 (The Cochrane Collaboration, Software Update, Oxford, UK) and STATA 14.0 (Stata Corporation, College Station, TX, USA) software. We pooled dichotomous data and continuous data with odds ratio and weighted mean difference (WMD), respectively, each with a corresponding 95% confidence interval (CI). A statistical significance was identified when *p* < 0.05. We performed the I^2^ test to determine the heterogeneity across the included studies. Regarding the inevitable heterogeneity, we used random-effect models for all the analyses. We also conducted sensitivity analyses by removing a study one-by-one to test whether the pooled results of remaining studies could be significantly changed [9].

## Results

### Study selection

**Fig. 1:**
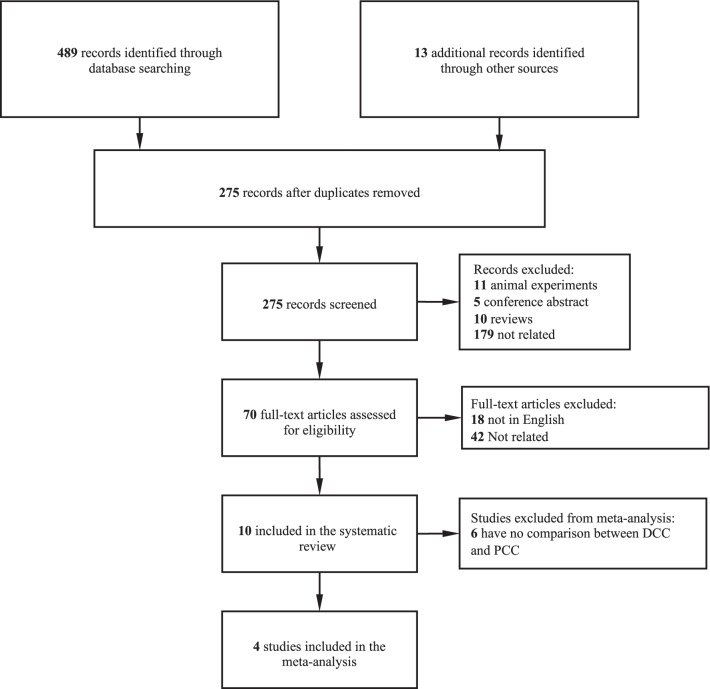
Flowchart of the selection process of this systematic review

Our literature search involved 275 studies. Ten studies were included in the systematic review [2, 4–6, 10–15]. Four retrospective observational studies [2, 4–6] with 211 patients with DCC and 1015 patients with PCC were finally included in the quantitative synthesis after carefully screening by 2 individual researchers (Fig. 1).

### Study characteristics and quality assessment

The basic characteristics of the included studies were presented in Table 1. All of them were single-center, retrospective observational studies conducted in the United States. Among these studies, the proportion of DCC after lung transplantation ranged from 6 to 29%. Rafiroiu and colleagues [5] performed 769 lung transplantations and 46 pairs of well-matched patients were generated using propensity score matching. A total of 211 patients who underwent DCC as well as 1015 patients who underwent PCC were enrolled in the analysis of post-transplant outcomes. More than 90% of the patients in these studies underwent bilateral transplantation, while the percentage was below 10% in the PCC cohort in the study of Shigemura and colleagues [4]. The quality of eligible studies was shown in Table 2. All the studies were of good quality according to NOS.

**Table 1:** Basic characteristics of included studies

Authors, year	Design	Country	Enrolled year	Quality	NOS	DCC/PCC cohorts, n	Bilateral lung Tx, DCC/PCC, n (%)	Indications for transplantation, n (%)	Type of incision	Indications for DCC, n (%)	Duration of DCC (days, median [range])
Force [6]	Single-center, retrospective	The United States	2003–2005	Good	7	7/21	7(100)/21(100)	DCC: COPD/A1AT, 1(14); CF, 1(14); IPF, 1(14); Sarcoidosis/PH, 3(43); Eisenmenger’s Syndrome, 1(14); PCC: COPD/A1AT, 13(62); CF/ bronchiectasis, 4(19); IPF, 4(19)	Bilateral anterior thoracotomy with or without sternal division^a^	based on cardiac and pulmonary impairment during attempted closure^a^	5.3 [3–7]
Shigemura [4]	Single-center, retrospective	The United States	2004–2011	Good	8	90/783	90(100)/75(10)	DCC: COPD, 8(9); IPF, 37(41); PPH, 20(22); scleroderma, 15(16); other, 11(12); PCC: COPD, 282(36); IPF, 149(19); PPH, 78(10); scleroderma, 47(6); other, 227(29)	NR	Acute lung edema, 40(44); oversized allografts, 38(42); coagulopathy, 29(32); hemodynamic instability, 18(20)	4.5(mean) [1–18]
Aguila [2]	Single-center, retrospective	The United States	2010–1014	Good	8	67/165	66(99)/161(98)	DCC: COPD, 3(4); ILD, 45(67); CF, 8(12); PH, 3(4); re-transplantation, 4(6); other, 4(6); PCC: COPD, 59(36); ILD, 59(36); CF, 33(20); PH, 4(2); re-transplantation, 4(2); other, 6(4)	bilateral, standard anterior thoracotomy; single, posterolateral thoracotomy^a^	Acute lung edema, oversized allografts, coagulopathy, hemodynamic instability^a^	2
Rafiroiu [5]	Single-center, retrospective	The United States	2009–2016	Good	7	46/46	44(96)/43(93)	DCC: IPF, 20(43); CF, 4(9); re-transplantation, 3(7); other, 19(41); PCC: IPF, 17(37); CF, 9(20); re-transplantation, 3(7); other, 17(37)	DCC: thoracotomy, 1(2); sternotomy, 32(70); clamshell, 13(28); PCC: thoracotomy, 6(13); sternotomy, 25(54); clamshell, 15(33)	Coagulopathy/bleeding, hemodynamic instability, hypoxia^a^	3 [2–6.2]

*COPD* chronic obstructive pulmonary disease/emphysema; *A1AT* alpha 1 antitrypsin deficiency; *CF* cystic fibrosis; *IPF* idiopathic pulmonary fibrosis; *PH* primary or secondary pulmonary hypertension; *PPH* primary pulmonary hypertension; *ILD* interstitial lung disease; *NR* non-reported

^a^Numbers of patients are not provided

**Table 2:** Quality assessment of included studies

Study	1	2	3	4	5A	5B	6	7	8
Force [6]	Yes	Yes	Yes	Yes	No	No	Yes	Yes	Yes
Shigemura [4]	Yes	Yes	Yes	Yes	Yes	No	Yes	Yes	Yes
Aguila [2]	Yes	Yes	Yes	Yes	Yes	No	Yes	Yes	Yes
Rafiroiu [5]	Yes	Yes	Yes	No	Yes	No	Yes	Yes	Yes

1, indicates exposed cohort truly representative; 2, non-exposed cohort drawn from the same community; 3, ascertainment of exposure; 4, outcome of interest not present at start; 5A, cohorts comparable on basis of age; 5B, cohorts comparable on other factor(s); 6, quality of outcome assessment; 7, follow-up long enough for outcomes to occur; and 8, complete accounting for cohorts

### Transplantation characteristics and risk factors for DCC

**Fig. 2:**
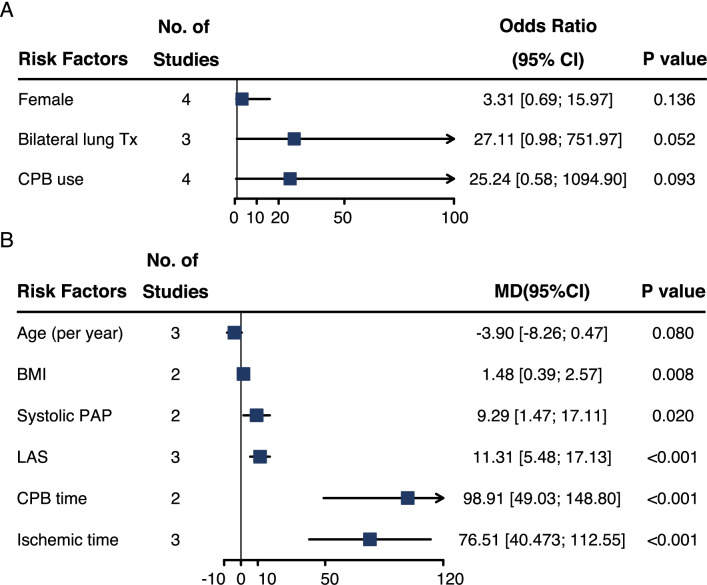
Forest plot summarizing meta-analysis of risk factors for DCC. **A** categorical variables; **B** continuous variables. CPB, cardiopulmonary bypass, BMI, body mass index, PAP, pulmonary arterial pressure, LAS, lung allocation score. OR, odds ratio. MD, mean difference

We first evaluated differences in transplantation characteristics between patients underwent DCC and PCC. The meta-analysis results were summarized in Fig. 2, which showed potential risk factors for DCC. Higher systolic pulmonary arterial pressure, higher lung allocation score, longer cardiopulmonary bypass duration and ischemic time were risk factors for DCC (Fig. 2).

### Primary outcome

#### Surgical site infection

**Fig. 3:**
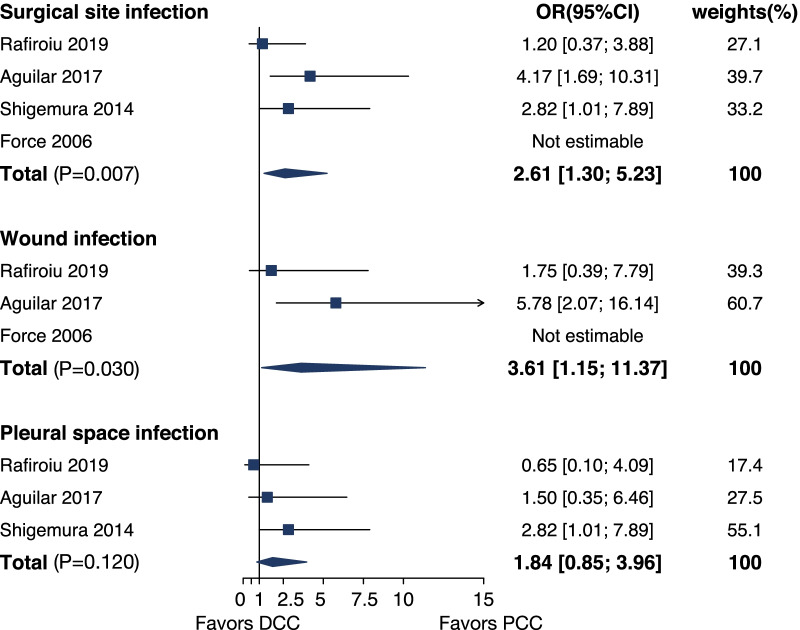
Forest plot for the incidence of surgical site infection, wound infection, pleural space infection for DCC versus PCC. OR, odds ratio

All the studies [2, 4–6] estimated the incidence of surgical site infection. The incidence of surgical site infections ranged from 0 to 19% in the DCC cohort and 0 to 13% in the PCC cohort. Pooled analysis showed an increased risk of surgical site infection in the DCC cohort [OR 2.61, 95%CI (1.30, 5.23), *p* = 0.007] (Fig. 3) compared with PCC cohort. It should be noted that pleural space infection was not included in the study of Force and colleagues [6]. and wound infection was not included in the study of Shigemura and colleagues [4]. Whereupon we performed subgroup analysis based on the site of infection. A similar result was found when comparing the incidence of wound infection between 2 cohorts [OR 3.61, 95%CI (1.15, 11.37), *p* = 0.030] (Fig. 3). And there was a trend toward an increased risk of pleural space infection associated with DCC, although it was not statistically significant [OR 1.84, 95%CI (0.85, 3.96), *p* = 0.120] (Fig. 3).

### Secondary outcomes

#### Primary graft dysfunction

All studies [2, 4–6] compared the incidence of PGD between the two groups after transplantation, all of which indicated that the incidence of severe PGD was higher in DCC cohort, although the pooled analysis was not conducted. Rafiroiu and colleagues [5] estimated postoperative PGD at 72 h. Aguila and colleagues [2] estimated that at the time of intensive care unit admission. Shigemura and colleagues [4] reported incidence of severe PGD requiring ECMO but neither grade nor the time point was provided. Force and colleagues [6] reported mean R-scores (i. e. PGD grades) of 2.14 and 0.48 in DCC and PCC cohorts, respectively.

#### Acute renal insufficiency

**Fig. 4:**
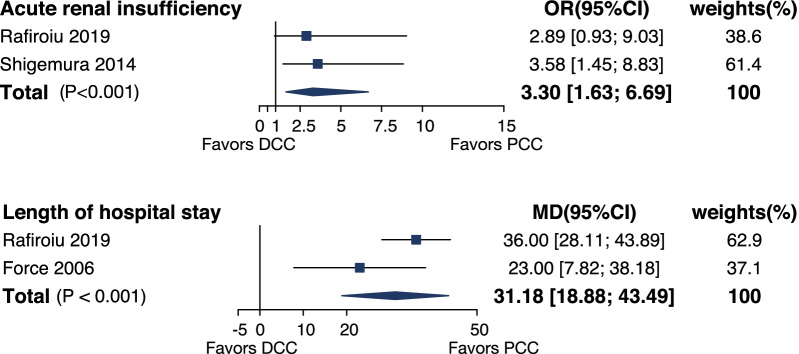
Forest plot for the acute renal insufficiency and length of hospital stay for DCC versus PCC. OR, odds ratio. MD, mean difference

Two studies [4, 5] investigated acute renal insufficiency. In the pooled analysis, the incidence was higher in DCC group [OR 3.30, 95%CI (1.63, 6.69), *p* < 0.001] (Fig. 4).

#### Postoperative ECMO use

We were unable to perform pooled analysis on this postoperative ECMO use. Shigemura and colleagues [4] reported 28 (31%) cases in patients underwent DCC, which was significantly higher than that in patients underwent PCC. Rafiroiu and colleagues [5] reported 11 (23%) cases in DCC group but did not make a comparison.

#### Length of hospital stays

The length of hospital stays was evaluated in 2 studies [5, 6]. Pooled analysis showed a longer length of hospital stay in DCC cohort [WMD 31.8, 95%CI (18.88, 43.49), *p* < 0.001] (Fig. 4).

#### Short- and long-term survival

**Fig. 5:**
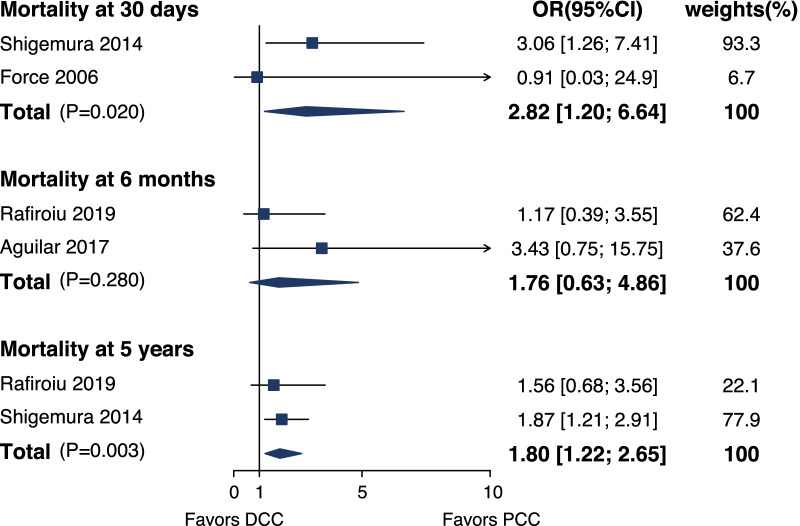
Forest plot for mortality at 30 days, 6 months, and 5 years after transplantation for DCC versus PCC. OR, odds ratio

All the studies [2, 4–6] evaluated the survival at different time points. Among all four studies, survival at 30 days, 90 days, 6 months, 1 year, and 5 years was reported. Shigemura and colleagues [4] reported that 30, 90 day, and 5 year mortality were higher in patients with DCC compared with the patients with PCC. Pooled analysis showed that mortality at 30 days was higher in the DCC cohort [OR 2.82, 95%CI (1.20, 6.64), *p* = 0.020] than that in the PCC cohort, and difference in survival at 6 months was not significant between 2 cohorts [OR 1.76, 95%CI (0.63, 4.86), *p* = 0.280] (Fig. 5). As for long-term survival, the 5 year survival of DCC patients ranged from 39 to 43%, and that of PCC patients ranged from 50 to 59%. Pooled analysis showed that DCC was associated with higher 5 year mortality [OR 1.80, 95%CI (1.22, 2.65), *p* = 0.003] (Fig. 5).

## Discussion

### Summary of findings

We conducted a systematic review and meta-analysis to identify the postoperative outcomes of DCC following lung transplantation. This was the first meta-analysis comparing postoperative outcomes between patients underwent DCC and PCC. We identified poorer post-transplant outcomes in DCC cohort including higher incidence of surgical site infection, PGD, acute renal insufficiency, prolonged length of hospital stays, and poorer short- and long-term overall survival except for 6 month survival.

DCC would be taken into consideration when patients facing the negative circumstances like profound graft edema, hemodynamic instability, coagulopathy/bleeding, impaired PaO_2_/FiO_2_ ratio, or oversized allografts [2, 4–6, 10–15], as early chest closure could increase the risk of airway resistance, atelectasis, and hemodynamic instability [16, 17]. It was reported that DCC also worked well on pediatric patients. Inoue and colleagues [14] reported 2 pediatric lung transplantations that combined DCC with ECMO. Both of the recipients survived and discharged on postoperative day 73 and 160, respectively. Chen and colleagues [12] reported single-lobe lung transplantation performed on a 6 year old girl, who survived well after 7 months. Another pediatric case was reported by Chang and colleagues [11], of which the chest was closed on post-operation day 2.

### Different DCC strategies

Techniques for DCC included (1) traditional skin closure with superficial structure sutured directly or sutured with supplementary medical bandage, and (2) newly introduced negative pressure wound therapy (NPWT) strategy. Shigemura and colleagues [4] reported 52 patients who underwent direct superficial structure closure with unapproximated ribs. Mohite and colleagues [15] introduced the technique of undermining the superficial structures on the chest wall with diathermy, utilizing on two cases with an oversized allograft. The oversized lungs were covered by the elongated skin with open intercostal spaces and disconnected sternal ends [15]. Both patients survived more than 1 year after surgery.

For patients whose skin cannot be sutured directly, a medical bandage or Bogota bag was required to cover the incision [5, 6], while such medical covers might bring some shortages. The cover might be sucked in during expiration and blown out during inspiration during mechanical ventilation, thus affecting airway pressure which was needed for a goal tidal volume [17]. And blood might stagnate under the cover and even leak out through the suture.

The unapproximated ribs accompanied with skin closure might require chest retractors left [4, 6]. Shigemura and colleagues [4] conducted subgroup analysis on postoperative outcomes regarding 3 different DCC strategies: (1) closed superficial structures with unapproximated ribs; (2) open superficial structures covered with medical bandage; (3) open superficial structures with chest retractors. They found patients with open superficial structures plus chest retractors resulted in highest incidence of severe PGD, respiratory complications, and mortality [4]. Subgroup analyses by Shigemura indicated carefully selection of different DCC strategies based on individual patient characteristics.

The approach of combining pleural packing and negative pressure wound therapy (NPWT) was introduced by Brioude and colleagues [10]. The purpose of pleural packing was to achieve a partial extrusion effect and avoid hemodynamic impact simultaneously [10]. With NPWT closure, a foam was placed under the chest wall with a chest tube and the wound was stuck to an adhesive film. This technique came out to be an effective approach to control coagulopathy and acute lung edema. Bakaeen and colleagues [18] showed that compared with latex membrane closure, the use of NPWT after cardiac surgery reduced reoperations for bleeding, transfusion, infection, and mortality. However, details of NPWT vary in different studies. For example, the setting of negative pressure ranged from 25 to 50 mmHg, continuously or discontinuously [5, 10]. We look forward to further reports or studies on detailed settings of NPWT in delayed chest closure.

### Infection

Infection was one of the most common causes of death after transplantation, accounting for up to 20% of all mortality [19, 20]. Patients after transplantation were at high risk for infection due to the immunosuppressive therapies [21]. Additionally, the disrupted physical barriers after transplantation, and the use of nasogastric and endotracheal tubes also increased the likelihood of aspiration and infection [22]. Extensive researches on open chest management after cardiac surgery also identified increased risk of hospital-acquired infections in the settings of ‘open chest’ [23–26].

We identified that the incidence of surgical site infection was higher in DCC group than that in PCC group. Shigemura and colleagues [4] evaluated the incidence of pleural space infection, while Aguilar and colleagues [2] studied both wound and pleural space infection. Both studies indicated DCC increased the incidence of surgical site infection. Rafiroiu and colleagues [5] suggested that aggressive antimicrobial prophylaxis might neutralize the infection risk. Aguilar and colleagues [2] modified their clinical practice by instituting antifungal prophylaxis in addition to broad-spectrum antibacterial antibiotics in patients requiring DCC, while they avoided DCC when possible in order to prevent the surgical site infection. The duration of DCC was also related to infection in patients requiring DCC. Aguilar and colleagues [2] suggest to minimize the length of DCC while in their cohort the median time to chest closure was 2 days after transplantation. Although avoiding DCC might be helpful to reduce the surgical site infection after transplantation, once DCC was unavoidable, antimicrobial prophylaxis or other aggressive supporting management to minimize DCC duration would be beneficial.

### Primary graft dysfunction

PGD contributed to the main postoperative morbidity and mortality after lung transplantation. This syndrome consisted of a series of manifestation of lung injury, including reperfusion edema, prolonged ventilator dependence, progressive hypoxemia. Ischemia or ischemia–reperfusion injury seemed to be the most important pathogenesis of PGD, which could cause alveolar epithelial injury, innate immune system activation, and increased permeability of pulmonary capillary bed [3, 27]. There was hypothesis that DCC might decrease the incidence of PGD, since it prevented increased intrathoracic and pericardial pressure from chest closure and avoided further insults to allografts [4]. Although we could not perform a pooled analysis, all included studies indicated that DCC was associated with an increased risk of PGD [2, 4–6]. In addition to DCC technique, it might be partly due to the subset of patients who underwent DCC were at the high risk for PGD after transplantation.

### Different managements other than DCC

In addition to DCC, extracorporeal membrane oxygenation (ECMO) was effective in managing PGD [28–30]. Both Shigemura [4] and Rafiroiu reported patients with DCC underwent ECMO to manage PGD. Inoue and colleagues [14] reported two cases who successfully treated PGD with veno-arterial ECMO via the thorax temporally closed with medical bandage. The veno-arterial ECMO could be conducted on patients with the combination of graft failure and inadequate cardiac performance, while the veno-venous ECMO could apply on patients with adequate cardiac function with PGD [30]. Management options also included inhaled nitric oxide or prostaglandin E1, which could improve gas exchange and reduce pulmonary vascular resistance [30–33]. Force and colleagues [6] reported to maintain patients with severe PGD on inhaled nitric oxide. Protective mechanical ventilation strategies, like low tidal volume ventilation and positive end-expiratory pressure support, were also beneficial for PGD [30, 34]. Fluid management also played an important role in post-transplant patients [30, 35]. Restrictive fluid management like appropriate diuresis, continuous renal replacement therapy, could help unload left ventricle and maintain hemodynamic stability [30, 35]. Meanwhile, we need to be cautious for hypovolemic state resulted from aggressively limited fluid management, which might lead to impaired tissue perfusion and graft injury [36].

Oversized donor lung was one of the major reasons for DCC. Increased perioperative complications and worse outcomes were observed in transplantation with significantly oversized allografts [37, 38]. In addition to DCC, size-reduced lung transplantation was increasingly performed when size mismatched between donor and recipient. The most common methods for volume reduction were peripheral segmental resection and wedge resection. Compared to standard lung transplantation, size-reduced lung transplantation for oversized grafts showed no difference in short-term outcomes [39–41] and long-term survival [42]. Lobar and split-lung transplant were also options other than DCC for over-sized grafts [38, 39].

### Limitations

This meta-analysis has several limitations. First, only 4 studies were eligible for quantitative synthesis. The limited number of studies restricted us to perform further analysis. Further studies covering more patients were warranted. Second, the heterogeneity among studies was significant. Strategies of DCC varied in different studies, although we conducted sensitivity analysis trying to evaluate the robustness. Third, the limited data restrict the subgroup analysis regarding single and bilateral lung transplants and different types of incision, which might contribute to differences in outcomes.

Patients who underwent DCC were already at an unfriendly circumstance. The pre- and peri-operative characteristics were incomparable between the DCC and PCC groups in the included studies, which might cause bias to the post-operative outcomes. Only Rafiroiu and colleagues [5] generated well-matched pairs through propensity score matching to account for the differences between the DCC and PCC cohorts, in which only the incidence of PGD and length of hospital stay were showed significant difference. The DCC should be applied with cautious assessment, whilst the balance between the benefits and disadvantages should be well considered. Furthermore, it could be helpful to identify risk factors which could influence the outcomes of DCC in the future studies.

## Conclusion

DCC was related to worse survival, increased postoperative infection, acute renal insufficiency, and prolonged length of hospital stay. It should be cautious to apply DCC after lung transplantation, nevertheless, DCC remains the life-saving step under dangerous circumstances. Further studies on the best options on DCC strategies and risk factors for its outcomes were warranted.

## Data Availability

The datasets used and/or analysed during the current study are available from the corresponding author on reasonable request.

## Abbreviations

DCC: Delayed chest closure
PCC: Primary chest closure
OR: Odds ratio
WMD: Weighted mean difference
PGD: Primary graft dysfunction
NOS: Newcastle–Ottawa quality assessment scale
CI: Confidence interval
EVLP: Ex vivo lung perfusion
NPWT: Negative pressure wound therapy
ECMO: Extracorporeal membrane oxygenation
